# “When there is no money, that is when I vomit blood”: the domino effect and the unfettered lethal exploitation of Black labor on Dominican sugar plantations

**DOI:** 10.1186/s12992-023-00963-4

**Published:** 2023-08-29

**Authors:** Brenda K. Wilson

**Affiliations:** 1 Global Health Program, University of California – San Diego, 9500 Gilman Dr, La Jolla, CA 92093 USA; 2 1527 1st Street Apt W307, Coronado, CA 92118 USA

**Keywords:** Plantationocene, Migrant farmworkers, Structural violence, Health inequalities, Sugar industry, Racial capitalism, Precarious employment, Transnational corporations, Imperialism, Haitian diaspora, Dominican Republic *bateyes*, Ethnography, Domino Sugar

## Abstract

**Background:**

In this article, I utilize the concept of the Plantationocene as an analytical framework to generate a holistic and historical understanding of the present-day struggles of a mostly Haitian migrant workforce on sugar plantations in the Dominican Republic.

**Methods:**

Inspired by Paul Farmer’s methodology, I combine political economy, history, and ethnography approaches to interpret the experiences of sugarcane cutters across historical and contemporary iterations of colonial, post-colonial, and neo-colonial practices over the course of five centuries.

**Results:**

My findings elucidate the enduring power of capitalism, implicating corporate and state elites, as the structural scaffolding for acts of racialized violence that condition the life-and-death circumstances of Black laborers on Caribbean plantations to this day. Although today’s sugarcane cutters may suffer differently than their enslaved or wage labor ancestors on the plantation, I argue that an unfettered racialized pattern of lethal exploitation is sustained through the structural violence of neoliberalism that links present conditions with the colonial past.

**Conclusions:**

Ultimately, this paper contributes understandings of the plantationocene’s enduring effects in the global south by demonstrating how imperialist arrangements of capitalism are not a distant memory from the colonial past but instead are present yet hidden and obscured while relocated and reanimated overseas to countries like the Dominican Republic, where American capitalists still exploit Black bodies for profit and power.

The story of a lump of sugar is a whole lesson in political economy, politics, and morality [1].

## Background

On June 19, 2016, Jean-Marc, a forty-year-old Haitian migrant farmworker, became dizzy and weak while cutting sugarcane on a Dominican plantation. Jean-Marc, like many cane cutters in the province of La Romana, was employed by the private sugar company, Central Romana Corporation [CR]. Much of CR’s mostly Black laborers (known as “cane cutters”) are unauthorized immigrants from Haiti, a circumstance that renders them vulnerable to racial exploitation and abuse. Cane cutters are paid poverty wages by CR and, on days like June 19, meant that Jean-Marc could not afford to sufficiently feed himself to sustain the grueling work demanded on the sugar plantation. Further, the denial of paid sick leave meant that he could not afford to take the day off, even though he was unwell. Consequently, he continued working instead of taking a break to rest his sick, malnourished body. Eventually he became so weak that he was no longer able to swing the machete. Jean-Marc began vomiting, experienced vision loss, and felt so ill that he decided to walk home. The distance back to his *batey*, the often-derelict residential areas where cane cutters and their families live on Dominican plantations, was four kilometers. Transportation is a luxury many cane cutters cannot afford, so he walked the distance, despite his rapidly worsening health. Less than sixty meters from his home, Jean-Marc collapsed. He fainted on the railroad tracks contracted by CR for transporting cane to the refinery in La Romana. A passing train ran over Jean-Marc, instantly killing him. His broken body was found many hours later; he left behind his wife and three young children. That Jean-Marc spent most of his brief adult life cutting cane in exchange for a cheap wage, gave years of his blood, sweat, and tears for capital and profits, and then, finally, gave his life by working himself *literally* to death to feed the American addiction for sugar and money is reflective of the structural violence of neoliberalism [2].

Dwelling in the “archipelago of Human Otherness” [3] Haitian sugarcane cutters like Jean-Marc belong to the racialized category of the politically and economically *damnés*, a term conceptualized by Frantz Fanon to describe the myriad ways the formerly colonized remain caught in the webs of colonialism [4]. As this study demonstrates, sugarcane cutters as *les damnés* embody the lethal consequences of today’s political economy – global neoliberal capitalism, – perpetuated through historical and contemporary forces and within a colonial and neocolonial framework [4]. The US and France amassed wealth by an unrelenting consumption of enslaved bodies and lands for sugar production; such extractive colonial economies created unequal power relations that are sustained today through exploitative neoliberal economies that continue Black suffering and killing on the plantation, exposing an ever-present imperialist mode of domination through globalized capitalism.

On modern-day plantations, abstract neoliberal practices of industry deregulation, private enterprise, free trade, corporate tax cuts, reduced government spending, and decreased labor power routinely exclude sugarcane cutters from a living wage, occupational protections, benefits, and collective bargaining. Haitian sugarcane cutters (like other migrant farmworkers around the world) represent precarious employment – a formation that has grown under neoliberal capitalism [5, 6]. Through framings of “illegality” [7, 8] cane cutters are devalued as needed but unwanted: needed insofar as their bodies are capable of producing sugar for capital and profit, but unwanted and denied rights and protections through the economies within which they work and live. Within this work environment, cane cutters are left vulnerable to economic insecurity and health consequences, unable to afford basic needs and die of preventable or treatable illness, far less is known about the tolls on mental and psychosocial health, and all of this points to the structural violence of neoliberal capitalism.

In the next section, I describe my methodology, an ethnographic bricolage that embraces the approach used by the late physician-anthropologist, Paul Farmer. I then present my findings that ultimately elucidate how the *longue durée* of capitalism provides the structural scaffolding for the continuation of racialized violence perpetrated against Black laborers on sugar plantations. This structurally violent political economy has produced and preserved an unequal and racialized power relation between labor and wealth – namely, an enduring imperialist mode of domination – that links present-day conditions with the colonial past. Although the political-economic modes and methods are different than the colonial and postcolonial eras, the consequences for Black bodies on sugar plantations today are the same, as illustrated through the neoliberal lived experiences of Haitian cane cutters. While today’s global sugar industry no longer relies on formal enslavement for cheap labor, the dehumanization and exploitation of Black bodies continues through the creation of transnational corporations, transformed relations between corporations and nation-states, and the deployment of neoliberal abstract practices. In this way, an unfettered pattern of racialized lethal exploitation through capitalism on sugar plantations has been carried forward to this day. I end my paper with some concluding reflections. This includes thoughts about the need for robust understandings of the plantationocene that center race/racism in analyses of present-day circumstances, and the need for structural interventions for a racially-just political economy in light of Black sugar laborers’ ongoing struggle against imperialism – an enduring legacy of the plantationocene that continues to configure relations between Global North-South today.

## Methods

### Conceptual framework and methodological approach

I apply the concept of the “plantationocene,” as a conceptual framework through which present-day experiences of Haitian migrant farmworkers on Dominican sugar plantations can be understood. Conceptualized by Donna Haraway and colleagues, the term “plantationocene” describes “the devastating transformation of diverse kinds of human-tended farms, pastures, and forests into extractive and enclosed plantations, relying on slave labor and other forms of exploited, alienated, and usually spatially transported labor.“ Contemporary plantationocene practices continue in globalized extractive industries, including sugar and other monocrop agrobusinesses. Using plantationocene as a lens facilitates a critical analysis across a space-time continuum for interpreting the continuing racialized struggles against imperialism and economic exploitation in the global south.

The findings in this paper focus on a subset of findings from a larger project [2], which represents an extension of Paul Farmer’s work in Haiti [9, 10] and expands on the Haitian diaspora in the DR. Methodologically, I incorporate Farmer’s approach of combining political economy and ethnography for generating holistic and historical understandings of health and inequalities. Such an ethnographic bricolage is essential for capturing the complexities and contexts of lived experience, structural inequalities, and power relations [12]. Farmer emphasized the need for understanding modern-day health disparities in historical and political-economic context, not merely for analytical clarity but also as a resistance to the unjust erasures of history and biology [13]. I consider Farmer’s approach as correspondent with Foucault’s “history of the present” [14] in which political investments of the body can be understood as borne out of historical origins and practices and yet maintained today by contemporary power structures that are similar but called by a different name. My paper works toward Farmer’s and Foucault’s calls for historical consciousness of modern-day bodily afflictions by combining political-economic and ethnographic approaches to interpret present-day experiences of sugarcane cutters within shifting iterations of colonial, post-colonial, and neo-colonial practices of capitalism. Emergent through my analysis is how the brutalities of colonialism have not ended but are continued today under the name of neoliberal globalization.

My political-economic analysis is positioned from an understanding of *material* relations of power as inseparable from understandings of the *discursive* frameworks that protect them from critical scrutiny. Although the structures of global capitalism are centered on the theories of political economy, its practices and policies became thinkable and actionable only through a discourse of racialization that produces subjects for a particular *mode* of political economy. In conceptualizing the “coloniality of power,” Anibal Quijano (2021) described how the racial axis of the political economy of capitalism has a colonial origin, character, and legacy. This colonial power structure was founded upon the idea of race and racial classification, a philosophy created by White Europeans during colonial expansion and deployed as a “new technique of domination/exploitation, in this case race/labor” [15]. Because racialized class divisions of labor and capital remain structurally intertwined, an accurate understanding of the historical and contemporary conditions of capitalism and its pathogenic effects on workers on the plantation requires an analysis of both race and economy.

To that extent, an understanding of the experiences of Haitian communities requires an understanding of *antihaitianismo*. I have argued elsewhere that *antihaitianismo* – an anti-Black discourse and set of practices deployed against Haitians in the DR [16] – was borne out of Imperial Spain’s ideas of *limpieza de sangre* (purity of blood) that provided an intellectual framework for a white supremacist justification for a racialized division of labor in new capitalist enterprises; *antihaitianismo* has continuously ensured an exploitable supply of Black bodies for sugar production and profit [2]. From the outset, *antihaitianismo* determined a capitalist division of labor that relegated Blacks at the bottom of the hierarchy and provided the logic for enslavement on the sugar plantations on the island of *Ayiti*, renamed by colonizers as Hispaniola. As such, the origins and practices of global capitalism, to which the sugar industry was fundamental, were made possible through the idea of *antihaitianismo*.

### Ethnographic data collection and analysis

To ensure that the research is rooted in the lived experiences of political economy, I draw on ethnographic work conducted in Dominican *bateyes* for a colonial and neocolonial exploration of the plantationocene processes and effects on Haitian sugarcane cutters. Semi-structured interviews with cane cutters and their partners living in *bateyes* located in the province of La Romana were collected in 2015 and 2016. Fifteen batey residents participated in the study, six males and nine females. In addition to formal interviews, I gathered information through informal group conversations with cane cutters and from local key informants (non-profits, clinical workers, community members). I conducted interviews alongside an interpreter, a female Kreyol-speaking Haitian native living in the DR who was knowledgeable in the local historical, cultural, and political contexts of Hispaniola. All interviews were approximately 120 min long and digitally recorded and transcribed verbatim. After each interview, field notes were recorded and transcribed describing context, non-verbal cues, rapport, and other observations.

Transcripts were uploaded into NVivo 10 software and, following a grounded theory approach, I conducted a line-by-line inductive analysis using open coding for themes and patterns to emerge. Thematic statements were selected and highlighted as representative of the lived experiences of study participants. I compared these statements to findings from the literature that addresses the structural and social determinants of health impacting migrant and minoritized populations.

### Ethics

Institutional research ethics approval was obtained from the University of Texas – Medical Branch ethics review board. At the time of this study, there was no local ethics review board in La Romana. Therefore, upon receiving approval at the Texas location, local host community approval was granted by the leadership of the Dominican Host Organization. Consent from all participants was obtained in the language of their preference.

## Results and discussion

My findings are organized by a tripartite division of analysis that historically traces political-economic practices from 1492 to the present. What emerges is how structural forces, past and present, have created and continue the logic of imperialism – in particular, a racialized division of labor and the lethal exploitation of Black bodies for sugar production and profit – into the twenty-first century.

### **Colonial capitalism (1492–1800): the production of *les damnés* on Hispaniola sugar plantations**

Contemporary plantationocene practices that continue the suffering and disparities for Haitian sugarcane cutters on Dominican plantations are historically rooted in the rise of early capitalism during European colonization. The island of Hispaniola is where Columbus first introduced *Saccharum officinarum* (sugarcane) to the western hemisphere, and where African slaves were first imported (Wright, 1916). For Imperial Spain, Columbus and his successors laid the foundations for one of the earliest capitalist endeavors: the global sugar industry, described by Sidney Mintz (citing Fernando Ortiz) as the “favored child of capitalism” (1985: 214), that relied on the creation of a racialized division of labor and wealth for its success. The origins of capitalism entailed violent practices, described by Karl Marx as “primitive accumulation” [18]. The primitive accumulation in Spain’s colony of Santo Domingo (present-day DR) and France’s colony of Saint-Domingue (present-day Haiti) first included dispossession of the indigenous *Taíno* population of their land, forced labor for mining and agriculture, and eventual annihilation. It also included the extraction and depletion of natural resources and the transfer of local wealth to Spain and France. The *Taíno*, estimated at one million in 1492, were decimated to approximately five hundred by 1548; to fill the void, primitive accumulation practices shifted toward the Transatlantic slave trade affecting the mass displacement and enslavement of Black bodies from Africa to sustain sugar production (*DR: First Colony*).

Christian Europe’s governing mentality of “empire” provided the justifying logic for the racialization of colonial-era capitalism, of which the sugar and slave trade enterprises were front and center. The logics of imperialism are rooted in the 1493 papal decree that granted rights to all lands in the western and southern hemispheres to Spain and Portugal; when Spain came to control significant portions, an unprecedented shift in the land-to-labor ratio [19] required new techniques to address concerns of an emerging merchant class and markets, including those in the colonies. In Santo Domingo and Saint-Domingue, mercantile economies were established and driven by the creation of a new *racialized* power structure – a hierarchical division of labor and wealth that imagined Black bodies at the bottom as dehumanized barbaric Other. Such ideas about white supremacy and inferiority of non-whites justified Black African enslavement and exploitation for the production of the luxury of sugar for satisfying the dietary desires of White Europeans, monetary desires of the emerging White capitalist classes, and political desires of Empire [17]. Herein was the violent and bloody creation of *les damnés* on Hispaniola and its lethal bodily effects, a key legacy of the plantationocene: Black slaves from Africa and generations of their creole descendants, whose bodies were *used* for sugar production, wealth, and empire until their bodies broke, fought back, or were no longer deemed *useful*, and then they were allowed to die or be killed.

The lethal exploitation of Black bodies and indigenous lands, enacted through this earliest iteration of capitalism, was visible from the outset, particularly in Saint-Domingue. French colonizers’ abuse of the landscape included large-scale deforestation for sugar plantations; the effects of French colonialism on Haiti’s environment are well-documented [20, 21]. Haiti provides an example of environmental racism, and today is considered the most environmentally degraded [22] and food insecure nation in the Western Hemisphere [23], and is among the top ten countries most at risk of climate change effects [20]. As the dehumanized, slaves were seen as exploitable and disposable and were commodified and consumed on plantations at a murderous rate by French colonizers. The slave death rate was so high and the birth rate so low that the French could not sustain their population without replenishment through importations from Africa [24]. The lethal exploitation of bodies and lands helped the colony earn its nickname, “Pearl of the Antilles,” for the vast wealth it generated for France, who by the late eighteenth century had forced more than half a million slaves to drive a booming sugar industry in Saint-Domingue [25].

Yet maintaining the racialized division of labor on plantations required new strategies when the absolute rule of imperials became increasingly challenged by planters and other capitalists, who believed they could intensify production and profit if the crown would impose less restrictions. Inspired by new “enlightened” ideas about the “natural rights” of Man, the bourgeoisie helped bring revolutionary ends to the *ancien régime* and forever altered terms of trade, which were reformulated through a different kind of govern*mentality* that desired a limited role of state power – i.e., liberalism [26]. Yet even as liberal thinkers extolled the virtues of liberty and equality of all *man*kind, the institution of slavery was expanded in Saint-Domingue, thereby exposing the paradox of the 1789 Declaration of the Rights of Man: only *White men* shall have recourse to human rights.

The realization that those rights did not apply to Blacks helped inspire the Haitian Revolution. In 1791, the slaves of Saint-Domingue proclaimed their humanity, right on the heels of the French and American Revolutions, fighting their way to liberation and independence in 1804, establishing Haiti as the world’s first Black Republic. Yet, the international reception of the newly established republics couldn’t have been more different. Haiti was economically punished by America and Europe for this transgression [11], and that punishment has continued to render the Haitian descendants of those slaves vulnerable to lethal exploitation through racialized capitalism, forcing them to migrate for work whether on shantyboats trying to reach the US or to the other side of the island looking for work on the sugar plantations.

From the beginning, the capitalist enterprise of sugar production relied on the production of *les damnés* through a racialized division of labor and wealth – practices that are embedded and furthered in the plantationocene. As I show in the following sections, although formal slavery – and its visibly evident strategies of lethal exploitation via colonial-era capitalism – were abolished across Hispaniola (1793 in Haiti; 1822 in the DR), the racialized labor hierarchy would be preserved through a new *kind* of imperialist mode of power. “Liberal” and “neoliberal” governmentalities created new capitalist strategies that – while less visibly evident than the tactics of empire in the colonies – were no less deadly and would ensure Black bodies would continue to suffer and die for sugar production and profit in both post-colonies.

### **Post-colonial liberal capitalism (1800–1970): the reproduction of *les damnés* on Hispaniola sugar plantations**

Nineteenth-century liberalism as a new “mentality of rule” abandoned the “megalomaniac and obsessive fantasy” of absolute rule [27] and addressed what emerging capitalists felt was too much state restriction on business. Liberalism became synonymous with laissez-faire economics, including ideas about “freeing” markets, and shaped the globalization of capitalism. Visions of market expansion and opportunities to amass wealth in the post-colonial Caribbean stirred foreign entrepreneurial imaginations; although Haiti and the DR were sovereign nations, foreign intervention continued. Two decades after Haitian independence, French warships returned and demanded that Haitians pay their former masters 150 million francs, an amount that was beyond the new republic’s capacity and has helped keep Haiti politically unstable and impoverished ever since [28]. Meanwhile, the DR heavily borrowed from European banks during the 1880-1890 s; on the brink of default, European warships arrived in Santo Domingo. In response, US president Teddy Roosevelt issued the Roosevelt Corollary of 1904 to the 1832 Monroe Doctrine under the rubric of “peacekeeping” and to prevent further colonization in the Western Hemisphere. These policies set the US on an interventionist path in the Latin America/Caribbean region, ushering in a *new* imperialism characterized by US domination and the creation of a transnational capitalist class and industries (like sugar).

An understanding of how power operates through transnational or multinational corporations is fundamental to understanding contemporary plantationocene effects in the global south. Between 1874 and 1916, Dominican presidents allied with foreign sugar planters, and a powerful White class was reconstituted – an early manifestation of a transnational capitalist class. The Basses, Vicinis, and other “sugar barons” helped resurrect a kingdom of sugar in the DR [29] and would amass fortunes through liberal capitalist practices, including tax exemptions and political concessions [30], monocultural economic development, and a steady supply of Black bodies for cheap labor. Through liberal capitalism, the imperial reach of the US in post-colonial Hispaniola was realized through not-so-discreet processes of “accumulation by dispossession” [31] that were evocative of the primitive accumulation of colonial capitalism. Dispossession occurred because foreign corporations, like the Spanish and French colonizers, wanted land and labor for sugar production and profit; and just like the imperialists before them, White sugar barons put those former slaves and their descendants – free but left destitute and desperate – back to work on sugar plantations for pitiful wages that kept them poor, sick, and marginalized.

By 1916, capitalist accumulation by dispossession was primarily carried out by American state and corporate actors [17, 32]. One set of techniques was the displacement of Haitian and Dominican peasants from their land and military occupation. In Haiti, transnational sugar companies like the Haitian-American Sugar Company poured into the countryside in 1911, forcing out the peasantry and resulting in regional instability. When the peasants fought back, US president Woodrow Wilson ordered a military invasion in April 1915. Meanwhile in the DR, American businessmen displaced small-scale farming with industrialized agriculture, driving rural farmers off their land in the process. Because of these interventions, the DR continued to struggle with debt and instability, inspiring Wilson to send marines in 1916 under the guise of negotiating the country’s struggles to repay its debt. Yet, with the rise of German influence and World War I coupled with American political *and* economic interests in securing its self-proclaimed “backyard,” Wilson authorized the military to fully take over. The US then had complete political control over Hispaniola, with the occupation of Haiti between 1915 and 1934 and the DR between 1916 and 1924.

American corporations took advantage of this new imperialist arrangement, and sugar companies were among the first to benefit. South Porto Rico Sugar Company (SPRSC), a New York-based multinational corporation who built the CR sugar mill in the DR in 1910, capitalized on the moment using violent tactics reminiscent of those used during the colonial era:

[T]he sugar companies’ methods were so efficient that they sometimes obtained titles to whole villages. In 1921, two hamlets, Caimoni and Higueral, which stood in the path of CR’s expanding fields, were burned to the ground. 150 families were left homeless, the company left no provisions for them [33].

In this unprecedented land steal, SPRSC accumulated 144,000 acres that displaced the local sugar farmers [34]. US conglomerates came to control more than 81% of total acreage under sugar cultivation; of the total tonnage produced, less than 5% was used by Dominicans, while more than 95% was exported to the US [33]. By the 1920s, American corporate interests dominated the Dominican sugar industry, making it a largely extractive economic enterprise for the US metropole. Additionally, like the colonizers before them, American planters contributed to environmental destruction through monocultural agriculture development.

A second, related set of capital accumulation by dispossession techniques was created to ensure a cheap labor supply, which exacerbated racial tensions between Haitians and Dominicans that continue to this day. Historically, the sugar industry on Hispaniola had relied on *slave labor*, but the end of formal slavery led capitalists to heavily rely on *migrant wage labor* – most of them Black and from Haiti – by the turn of the century [35], thereby reproducing the racialized division of labor and wealth like the colonizers before them. American sugar businessmen petitioned the US military-backed government, arguing that an imported labor source was necessary because too few Dominicans were available [36]. To the dismay of Dominican nationals [16], the sugar businessmen’s demands were met when they were allowed to import Haitians to Dominican plantations – a convenient yet highly exploitative maneuver, given that Haiti was also under American occupation and the recent dispossession of Haitian peasants from their lands and livelihoods had effectively produced an exploitable population in desperate need of work. This new imperialist mode of domination was exercised through transnational arrangements that superseded Haitian and Dominican sovereignty and borders, allowing American sugar companies to keep labor costs down and aggressively amass profits from intensifying production in the DR, while exploiting the surplus of displaced peasants in Haiti that the US had created. An estimated 10,000 Haitians per year were brought to the DR; they often ended up in the same place that their slave ancestors had fought to escape: the sugar plantation [37]. Unlike their slave ancestors, wage laborers were paid but received a pittance of less than a dollar a day, an insufficient amount for survival even then [33].

The lethal exploitation on Black bodies, now enacted through liberal capitalism of the postcolonial era, can also be understood in relation to the corporate neglect of sugarcane cutters’ living conditions. Cane cutters were housed in extremely impoverished developments called *bateyes*, set up by sugar companies and poorly constructed, overcrowded, and lacked electricity, running water, and plumbing. In 1926, a US consul in the DR reported that conditions were “primitive in the extreme” and that most laborers “exist solely on a diet of yams, bananas, and other fruits, the average expenditure for food being estimated at from 15 to 20 cents per day.” Men and women wore cheap clothing until they were useless, and “the children for a considerable number of years are devoid of clothing” [33]. Residents made a “kind of slipper … made of disused automobile tires, which costs them nothing” (ibid.). American missionaries and students who visit the *bateyes* today, largely ignorant of this history [2], would notice that conditions have not significantly changed in nearly one hundred years.

Neither the US military-backed government nor subsequent Dominican governments improved the circumstances in which sugarcane cutters lived and worked [36]. Both governments found “new and creative ways of siphoning money even from the pittance the workers were paid,” including wages promised to them after they finished the harvest but that they never received [38]. Basic needs were unmet because industrialization had rested on a system of family monopolies that took advantage of developments to amass enormous savings that were transferred outside the country [39] or invested for foreign tourists. For example, rather than invest in the betterment of cane cutters by providing a living wage or improving *bateyes*, the owner of the CR mill during the 1970s – the US multinational conglomerate, Gulf & Western – instead invested profits into building a playground for the world’s rich and famous, a five-star luxury resort known as Casa de Campo.

Such American capitalist accumulation by dispossession practices had long-lasting, destructive effects on populations in both nations of the bifurcated island. These included [1] economically, the transfer of the domestic economy and wealth into foreign hands and increased dependence on international markets; [2] socio-politically, worsened social unrest in the countryside, with violence that rippled into urban areas; [3] ecologically, deforestation and destruction of diversified agriculture, and failures of monoculture plantation economies to produce a local food supply; and, [4] socio-culturally, exacerbated racial resentment between Haitians and Dominicans. The rural peasantry, fearing further loss of land to US corporations, took an anti-imperialist stance and resisted American occupiers through guerrilla warfare, often resulting in brutal suppression.

Under benevolent framings of US protection and development of postcolonial Hispaniola, an imperialist power structure was maintained through state and corporate practices of liberal capitalism, which institutionalized racism and reproduced Haitian cane cutters as *les damnés* by wiring in and preserving the racialized division of labor and wealth from the colonial era. Racialized primitive accumulation is therefore not an historical relic of colonial-era capitalism but is an ongoing feature of modern liberal capitalism as practiced through the plantationocene. Its success is owed not merely to the political realm of state power but also to the economic realm of corporate power, whose bourgeois interests commanded state policy and military suppression of brown and black bodies. Indeed, many liberal capitalists became proud imperialists during the twentieth century; Hannah Arendt’s interpretation of this new imperialism as “the first stage in political rule of the bourgeoisie” seems accurate [40].

In the next section I describe how the wealthy owners of CR have carried forward the racialized division of labor and wealth from the colonial and postcolonial eras through today’s political economy: neoliberal capitalism. Neoliberalism, a *neo-* variant of liberal capitalism, is a new political-economic reconfiguration – often subsumed under the term globalization – that is a set of economic policies conceptualized by the Washington Consensus, driven by the global north, and imposed on much of the world including debt-ridden postcolonial states in the global south [41]. In essence, neoliberalism is a political project that has achieved through transformative actions the atomization of workers and the destruction of collective structures (like trade unions for the defense of workers’ rights) perceived as obstructions to the ideal of a perfect “free” market [42]. Globalized neoliberal capitalism, embedded with layers of exploitative practices, has protected and enriched transnational corporations like CR, whose owners have amassed billions of dollars in profits off of the backs of exploited Black labor. My analysis below explores the lived experiences of contemporary Haitian sugarcane cutters to understand the effects of neoliberal capitalism on their health and lives. Neoliberal capitalism, through abstract and immaterial practices but with concretized and materialized effects on bodies, provides the structural scaffolding that sustains Haitian cane cutters as *les damnés* on the contemporary plantation, with lethal effects evocative of those seen during colonial-era capitalism. This represents the plantationocene’s long duration into the twenty-first century across different forms of capital and racial organization.

### **The neo-coloniality of neoliberal capitalism (1980s-present): sustaining the presence of *les damnés *today**

#### Lived experiences of neocolonial capitalism

When I first met him, Josef (age 64) was sitting under a tree with his bandaged foot propped up on a rock in the *batey*. He was employed as a cane cutter by CR in the 1980s after crossing the border from Haiti. I asked about his injury, and he told me that it was a compound fracture that “won’t heal for some reason,” but the company’s failure to provide proper hygiene and sanitation infrastructure in the *batey* arguably had something to do with it. “Cane-cutting is exhausting work,” Josef told me. “We cut cane, load it onto carts, take it to the weighing station. We get a lunch break, but I never have enough food to feel strong enough.” Cane cutters earn their wage based on daily tonnage; they told me that, on average, a young healthy man could harvest three or four tons of sugarcane daily, earning him approximately 500–700 Dominican pesos per day ($10–15), an amount well below a living wage. The poverty wage doled out by CR perpetuates illness and hunger, two ailments that expose sugarcane cutters to further illness, injury, and even death.

“It’s underpaid and dangerous work,” Josef noted. “I have seen workers getting cut badly with machetes or injured by flying shards of cane. Sometimes my blood pressure gets so high that I vomit blood. I have medication, but I frequently run out of it. When there is no money for medication, that’s when I vomit blood in the sugar fields.” Cane cutters work for an industry known for its hazardous working conditions. According to one report, 345 workers (47%) surveyed had been injured or become ill during their employment in the sugar sector, with many receiving deficient medical care [43]. Using machetes to harvest cane is back-breaking, bloody, and dangerous work not only because of the injuries that may occur but also the physical and mental exhaustion from working prolonged hours in an intense Caribbean climate [44].

In addition to medical injury, Josef touched on the injuriousness of racialized pay devaluation within this work environment:

The Haitians, who are the ones that do the hard work, are the ones who are the least paid. We are undervalued *because* we are Haitian. The company won’t raise our wages; instead, they find ways to *decrease* it. If you cut five cartloads, then the *pesadores* [company men in charge of the scale] only reports two and records the rest under a different name, either pocketing that amount for himself or giving it to a friend. That’s how they steal from cane cutters’ work every day.

Poverty wages are reinforced at the weighing station, where cane cutters are paid based on the tonnage of cane that is cut rather than by number of hours worked, reflecting a racially discriminatory pay system. In this way, cane cutters are doubly exploited – denied a living wage for each ton that they cut and further disadvantaged at the weighing station. Cheating cane cutters at the scale was identified as a problem in the early 1990s and was temporarily reduced in 1996 when labor unions required official inspection. However, the globalization of neoliberal policies has weakened trade union power that had afforded at least some degree of protection for cane cutters. Deunionization resulted in the return of wage exploitation at the scale and other violations of labor rights. A 2013 report found evidence of the retaliatory firings of workers for affiliation with unions or for attempts to unionize; therefore, it seems that little, if anything, has been done to enforce fair wage payments at the weighing scales [45].

In addition to poverty wages and weakened collective bargaining power, sugarcane cutters are disadvantaged through the denial of pensions. As a critically ill man living with HIV, Jonas Pierre (age 59) is no longer able to work and at the mercy of the other residents in his *batey* for food and assistance:

My house is leaking; the rain falls on my bed that is now rotten. I cannot do anything about it. I cannot move out of this *batey* because I would have to pay for housing elsewhere, but I cannot afford to move because I am too sick to work … If you are here legally, then you are eligible for a pension. But that doesn’t mean anything because I don’t know anyone at age 60 who has actually received their pension.

Elderly migrant sugarcane cutters without proper legal documentation are denied pensions and other benefits, even if their work was equal to that of legal residents and citizens. However, as Jonas Pierre pointed out, even those *with* legal documents are not guaranteed a pension.

Ethnic Haitian cane cutters, even if they were born in the DR, are denied pensions upon retirement by CR through racialized tactics, as the story of Jacques revealed. Three years ago, Jacques (age 69) injured his leg while working, which left him permanently unable to walk and work. Since he was of retirement age, his boss promised him a pension on the condition that he provided proof of legal residency. When Jacques showed his Dominican birth certificate, the company administrator noticed that the spelling of his name did not exactly match that on his employee records: one letter was missing. Based on that technicality the company denied Jacques his pension. This happens to employees with Haitian-Creole names (e.g., the Haitian name “Jacques” was misspelled by Dominican Spanish-speakers as “Jacque”). Employers will accidentally or purposely misspell the names of darker-skinned employees – those who *look* Haitian – an action that can be later used to deny those workers their rights to equal wages, medical benefits, and pensions. Jacques noted the bitter irony: “But the company didn’t bother asking me for my papers upon hiring me! That’s probably when my name was misspelled in their records. It was only *after* I got injured that they cared about legal matters!” This finding makes evident that CR’s practice of excluding cane cutters from rights and protections is based on race, not legality, given that those corporate abuses target those who appear Haitian (i.e., African physiques, French-Creole cultural traits).

All the above reveals the structural violence of neoliberal capitalism, as evidenced through cane cutters’ lived experiences. The neoliberal practices of racialized pay devaluation, poverty wages, minimal occupational protections, deunionization, denial of pensions and benefits, and constant threat of deportation if they resist those terms have life-and-death consequences for the sugar industry’s mostly Black cane cutters and is indicative of the persistence of racialized capitalism. Such abstract practices characteristic of neoliberal capitalism are less visible and hidden from public consciousness and scrutiny as compared to those seen during capitalism of the colonial- and postcolonial-eras, yet the lethal exploitation of sugarcane cutters continues, as the experiences of Jacques, Jonas Pierre, Josef, and Jean-Marc revealed. Cane cutters’ enduring presence as *les damnés* is a function of today’s neoliberal political economy and legacy of the plantationocene.

#### Neocolonial capitalism as structural violence

What other neoliberal capitalist accumulation by dispossession strategies are deployed by transnational corporations like CR? One strategy includes corporate tax breaks in the US, which help facilitate exploitation on sugar plantations in both the US and DR. Since 1985, CR has been under the ownership of the children of Alfonso Fanjul, Sr., who was born into the colonial aristocracy in Havana and presided over one of the largest sugar holdings in Cuba. The Fanjul family fled Fidel Castro’s regime and resettled in Palm Beach, Florida, and with the help of incentives handed out from the US government and taxpayers, the Fanjuls – like their European colonial predecessors – commenced accumulating capital by dispossession by exploiting both land and labor: they bought out and displaced scores of American farmers, drained thousands of acres of the Everglades swamp to create 180,000 acres of sugarcane fields, and then imported a Black poor Caribbean workforce to keep costs down [29]. In the 1980s, Alfonso Fanjul, Jr., a permanent American resident, and his brother Jose, an American citizen, became chairman and vice chairman of Florida Crystals, the majority owner of Domino Sugar. They expanded their acreage by acquiring SPRSC from Gulf & Western Industries, renaming it Central Romana Corporation.

A second, related technique is political campaign donations, which helped further exploitation by paying state officials to ignore their own neoliberal free trade policies like the Central American Free Trade Agreement [CAFTA-DR]. The Fanjuls have increasingly blurred the lines between political/public and economic/private spheres by integrating into political circles in the US, especially through the heavy political campaign donations they give to both sides of the political spectrum including the Bushes, Clintons, and Marco Rubio [46–48]. The Fanjuls have amassed wealth through other highly profitable operations in the US and DR. According to one report,

In the DR, CR pays some of the lowest wages in the country, produces most of the country’s allotment of sugar exported to the US and, thanks to CAFTA-DR, and pays dwindling tariffs for those exports; in the US, they sell their sugar at sometimes two to three times the global market price, thanks to import limits and price supports [46].

Under the terms of CAFTA-DR, which the DR joined in 2004 for the creation of free trade areas through the reduction of tariffs, the US government slowly increased the amount of sugar imports from the DR – the majority of which came from CR. The continuation of a racialized division of labor and the lethal exploitation of Black bodies on modern-day sugar plantations is facilitated through US government provisions of corporate subsidies and tax breaks for wealthy executives and corporations [49], through political campaign donations that incentivize Washington to turn a blind eye to trade policies and labor protections, and through American voters (whether knowingly or not) that back those state officials and who are unconscious to the distorting effects of political campaign contributions and its public health effects [50].

A third set of neoliberal strategies have weakened labor power and include de-unionization, privatization, and deregulation. In 1988, Dominican labor unions called for a general strike to protest work conditions and salaries, but Dominican president Balaguer, who embraced a neoliberal distaste for organized labor, sent armed forces to suppress it [39]. The following year, hundreds of farmworkers took to the streets and clashed with the army. Despite labor rights to collective bargaining, the fear of repercussions if they attempt to collectivize and the threat of deportation is always overhead for Haitian laborers.

Privatization and deregulation of the sugar industry took shape in the 1990s. Even though the living and working conditions for sugarcane cutters were deplorable under public/state ownership as described in the previous section, the privatization of industry has not guaranteed better. CR had already demonstrated for a century how the private sector can abuse and exploit its racialized workforce and how the logic of “private property” blocks access from outside scrutiny. Relatedly, workers’ protections were eroded through deregulation, which transfers responsibility from the state to corporations to monitor their own impacts on workers, effectively undercutting many of the international policies that exist to increase labor and health standards. Occupational safety concerns have been documented by other studies, including one that found that cane cutters were not typically given protective clothing like goggles, boots, and gloves [51]. Coupled with union-busting efforts, sugarcane cutters have little power to negotiate to improve their life circumstances. CAFTA-DR included a set of labor laws for improving the working conditions of cane cutters; despite the presence of international protections, little has been done to enforce them. In 2013, a US Department of Labor report cited violations of labor policies, including minimum wage, working hours, overtime pay, occupational safety and health, prohibition of forced labor, racial discrimination, and retaliatory firings of workers for attempts to unionize [45]. Although the report indicted the Vicini-owned operations in a neighboring province, similar abuses are reported elsewhere on the Fanjuls’ CR [29, 46].

Today, CR is the largest private producer of sugar in the DR and the US is the largest importer of Dominican sugar, the bulk of which comes from the Fanjuls’ Dominican subsidiary [52] and is produced off the backs of exploited Black Haitian labor – extending and refracting a colonial legacy. The reproduction and enduring presence of *les damnés* is made possible through abstract neoliberal capitalist practices of deregulation, privatization, de-unionization, government subsidies and tax breaks for corporations, and corporate donations to political campaigns. All the above reflects contemporaneous practices that embed the plantationocene – practices that have sustained racialized capitalism and the lethal exploitation of Black bodies on sugar plantations, thereby exposing the imperialist reach of the US in Hispaniola to this day. Implicated in this structural violence are American state and corporate elites as well as average American citizens, the primary consumers of the Dominican sugar harvested by exploited Black labor. The lethal exploitation perpetuated against cane cutters traps them in a state of poverty and illness, that sometimes leads to premature or preventable death, and is driven by our mass consumption of the luxury of sugar, with all its affiliated health ills, while the Fanjuls amass billions in profit annually and their socialite daughters can sit on the boards (ironically) of prestigious American cancer research institutes [53]. Meanwhile, American tourists, university students, and medical missionaries enjoy CR’s Casa de Campo luxury resort – a privilege well beyond the reach of sugarcane cutters, yet made possible through the blood, sweat, and tears of their exploited labor ever-perpetually on the sugar plantation.

## Conclusion

For the “1619 Project,” Matthew Desmond posited that when one starts with the plantation – a space that produced a booming economy – one can begin to understand the brutality of American capitalism, characterized by America’s reliance on violence exerted against non-Whites for its creation and reproduction. He wrote:

The cotton plantation was America’s first big business, and the nation’s first corporate Big Brother was the overseer. And behind every cold calculation, every rational fine-tuning of the system, violence lurked. Plantation owners used a combination of incentives and punishments to squeeze as much as possible out of enslaved workers. Some beaten workers passed out from the pain and woke up vomiting. Some “danced” or “trembled” with every hit. An 1829 first-person account from Alabama recorded an overseer’s shoving the faces of women he thought had picked too slow into their cotton baskets and opening up their backs. To the historian Edward Baptist, before the Civil War, Americans “lived in an economy whose bottom gear was torture.” [54].

My study found that although today’s Black laborers on plantations may suffer differently than their enslaved or wage labor ancestors, an unfettered racialized pattern of lethal exploitation on plantations persists through an imperialist logic of neoliberal capitalism that is carried forward from the colonial and postcolonial eras. While today’s global sugar industry no longer relies on formal enslavement for cheap labor, it continues to dehumanize and exploit Black bodies through neoliberal abstract practices that are deployed by wealthy owners of transnational corporations like CR for maximizing profit and power. The imperialist domination of Haitian bodies and their preservation as *les damnés* on contemporary sugar plantations is not carried out visibly and violently “at the point of the bayonet or under cannon fire” [4] but instead is continued through the transnational power structures of invisible, yet no less violent, repressive neoliberal capitalism.

The invisible forces and effects of structural violence are made visible through ethnographic research. Cane cutters’ stories reflect how the embodied effects of structural violence manifest as unequal power and unequal life chances; relegated as *les damnés*, they struggle on an everyday basis against not only a corporeal death but also a political-economic death – they must resist biological pathologies and “pathologies of power” [55]. Despite neoliberal rhetorics of freedom (of markets, trade, individuals) [56], the realities of neoliberal capitalism for Haitian cane cutters – and other dispossessed and displaced poor of the global south – is that they are dying for economic growth, just as they bled for liberal capitalism during the industrial revolution of the nineteenth century, and just as they bled for colonial capitalism of the fifteenth through eighteenth centuries [1, 9, 57–60].

The plantationocene’s enduring effects in the global south demonstrate how imperialist arrangements through capitalism are not a distant memory from our colonial past but instead are present yet hidden, relocated, and reanimated overseas to countries like the DR, where American capitalists, through transnational processes, still exploit Black bodies for profit and power. This exposes the neo-coloniality of globalized neoliberal capitalism, discernable in the creative and subversive ways the racialized economies of imperialism are sustained and reinforced. Neoliberal reconfigurations of time-space helped project US economic power outwards, forcing open markets overseas and creating opportunities to proliferate monopoly powers in the form of *transnational* corporate operations, with social and political repercussions for the global poor [61]. Although worker protections were not eliminated through neoliberalism, these processes increased the pool of disposable workers and, subsequently, undercut their ability to demand workplace protections [41]. These and other invisible “killing abstractions” [62, 63] of neoliberalism produce material (if not always visible) effects, including the disposability, suffering, and inequality of Black bodies on sugar plantations. This ongoing consumption of disposable bodies for sugar production and profit reminds me of what bell hooks described as “eating the other” [64], a process characteristic of a White patriarchal capitalist system that reduces dark-skinned Others to a commodity and reproduces racialized class power, wealth, and privilege across time and space. Such a deadly politics of consumption through extractive capitalist economies persists to this day even though this labor is no longer based on the formal enslavement of Black bodies.

Critical research investigations that use the concept of plantationocene to understand present-day health and social disparities in the postcolonial global south, like Hispaniola, help advance how we think about the root causes of illness and poverty and how we think about actions for repair and redress. I agree with others that a robust and accurate thinking with the “plantationocene” requires greater attention to race and colonialism – one that analyzes the intermeshing colonialist, racialist, and capitalist structuring of modern-day life since the 15th century [65, 66]. Although not explored in depth in this paper, I propose that such scholarship be applied to advance conversations around reparation, particularly in the localities of racialized descendants of American and European colonialism and transatlantic slavery. Such discussions might deliberate on what widespread reparations could look like and what extent reparations would be capable of both enacting repair and tackling inequality.

The unrelenting persistence of lethal exploitation of Black Haitian bodies for labor and profit merits a more deliberate public and political discussion around decolonization and the ways that reparations are an integral part of that project. That Germany, a colonial power, has formally recognized genocide and other crimes of German colonial rule in Namibia and has committed €1.1 billion for reconstruction and development to support Namibia is a promising sign. Although too early to tell, perhaps this move may pave the way for other colonial powers to do the same. In the case of Haitians and the Haitian diaspora, France and the US owe a great debt to Haiti for the resources, wealth, and power that was amassed by exploiting bodies and lands for centuries. A serious reckoning and redress of the crimes of colonial rule – including “every drop of their sweat and blood, and each parcel of land” [67] taken and transferred into wealth for imperial centers – is warranted and is part of the dismantling of the enduring effects of the plantationocene. Arguments for historic justice via reparation dovetail with the perspectives of Haitian scholar Michel-Rolph Trouillet who argued that Haitian predicaments are not due to ignorance but to foreign hegemony, that capitalism is not invincible, and that we “can envision a future that is not driven by sheer accumulation” and “where one race does not dominate another” [68].

## Data Availability

The datasets during and/or analyzed during the current study available from the corresponding author on reasonable request.

## Abbreviations

CAFTA-DR: Central American Free Trade AgreementDR
CR: Central Romana Corporation
DR: Dominican Republic
US: United States
